# Effect of Au Plasmonic Material on Poly M-Toluidine for Photoelectrochemical Hydrogen Generation from Sewage Water

**DOI:** 10.3390/polym14040768

**Published:** 2022-02-16

**Authors:** Ahmed Adel A. Abdelazeez, N.M.A. Hadia, Abdel-Hamid I. Mourad, Gehad Abd El-Fatah, Mohamed Shaban, Ashour M. Ahmed, Meshal Alzaid, Nizamudeen Cherupurakal, Mohamed Rabia

**Affiliations:** 1 Nanoscale Science, Chemistry Department, University of North Carolina at Charlotte, Charlotte, NC 28223, USA; aabdelh2@uncc.edu; 2 Physics Department, College of Science, Jouf University, Al-Jouf, Sakaka P.O. Box 2014, Saudi Arabia; mmalzaid@ju.edu.sa; 3 Basic Sciences Research Unit, Jouf University, Sakaka P.O. Box 2014, Saudi Arabia; 4 Mechanical and Aerospace Engineering Department, College of Engineering, United Arab Emirate University, Al Ain 15551, United Arab Emirates; 201890121@uaeu.ac.ae; 5 Mechanical Design Department, Faculty of Engineering, Helwan University, Cairo 11795, Egypt; 6 Nanomaterials Science Research Laboratory, Chemistry Department, Faculty of Science, Beni-Suef University, Beni Suef 62514, Egypt; gehad.abdelfattah1508@science.bsu.edu.eg (G.A.E.-F.); mohamedchem@science.bsu.edu.eg (M.R.); 7 Nanophotonics and Applications Lab, Physics Department, Faculty of Science, Beni-Suef University, Beni Suef 62514, Egypt; mssfadel@aucegypt.edu (M.S.); ashour.elshemey@gmail.com (A.M.A.); 8 Physics Department, Faculty of Science, Islamic University of Madinah, P.O. Box 170, Madinah 42351, Saudi Arabia

**Keywords:** poly(m toluidine), photocatalyst, water splitting, photoelectrochemical H_2_ generation, plasmonic Au nanoparticles, sewage water

## Abstract

This study provides H_2_ gas as a renewable energy source from sewage water splitting reaction using a PMT/Au photocathode. So, this study has a dual benefit for hydrogen generation; at the same time, it removes the contaminations of sewage water. The preparation of the PMT is carried out through the polymerization process from an acid medium. Then, the Au sputter was carried out using the sputter device under different times (1 and 2 min) for PMT/Au-1 min and PMT/Au-2min, respectively. The complete analyses confirm the chemical structure, such as XRD, FTIR, HNMR, SEM, and Vis-UV optical analyses. The prepared electrode PMT/Au is used for the hydrogen generation reaction using Na_2_S_2_O_3_ or sewage water as an electrolyte. The PMT crystalline size is 15 nm. The incident photon to current efficiency (*IPCE*) efficiency increases from 2.3 to 3.6% (at 390 nm), and the number of H_2_ moles increases from 8.4 to 33.1 mmol h^−1^ cm^−2^ for using Na_2_S_2_O_3_ and sewage water as electrolyte, respectively. Moreover, all the thermodynamic parameters, such as activation energy (*E_a_*), enthalpy (ΔH*), and entropy (ΔS*), were calculated; additionally, a simple mechanism is mentioned for the water-splitting reaction.

## 1. Introduction

Fossil fuels, such as petroleum, oil, and natural gas, represent an essential energy source. The leakage of these sources and the contaminations of gases exhausts, SOx, NOx, and Cox, push scientists worldwide to think about a replaceable energy source, which is represented by renewable energy sources [1,2,3]. These renewable energy sources have advantages represented in clean energy and sustainability [4,5,6].

Hydrogen gas represents the primary solar energy source produced under the water-splitting reaction [7,8]. Hydrogen gas has advantages in its high combustion and performance [9]. Moreover, water splitting is carried out using photocatalytic materials under the light, thus decreasing the voltage used for this reaction. The photocatalytic materials must be semiconductor materials that can form electron–hole pairs, in which the electrons are form to represent the produced current density values. With the increase in the surface area, the produced J_ph_ values increases. So, the photocatalytic materials with nanowires, nanotubes, and nanosheets have the optimum properties for the hydrogen generation process [10]. Although metal oxides, sulfide, and nitrides represent suitable catalytic materials, low hydrogen rates and complex preparation methods limit the use of such semiconductor materials [11,12,13,14].

The effect of noble metals, such as Pt, Rh, and Au, on semiconductor materials for hydrogen generation was studied by different researchers [15]. These noble metals have a plasmonic effect that causes photon capture and electron resonance on the neighbor semiconductor materials [16]. Shi et al. [17] studied the CdS/Pt photocatalytic reaction for hydrogen generation using a Xe lamp. Zhao et al. [18] studied the TiO_2_-Au-CdS photocatalyst reaction under 300 W Xe, in which the Au nanomaterials activate the surface of CdS and TiO2 and cause energy transfer between them.

Polymeric nanomaterials, such as aniline and its derivatives, are replaceable sources of materials with high advantages, especially their easy preparation, low cost, high stability, and reproducibility [19,20]. Moreover, these polymer materials have great optical properties that qualify them as high efficient photocatalytic materials [21,22].

There have been some studies carried out on these polymer materials for photocatalytic applications and some methods have been proposed for preparing photocatalytic polymers, such as chemical, electrochemical, and electrospun methods [23,24,25,26].

Ghosh et al. [27] studied thiophene polymers (poly(3,4-ethylene dioxythiophene)) as catalytic materials for contamination decomposition. Yin et al. [28] studied poly(diphenyl butadiyne) for pollution removal from fiber materials. Ramohlola et al. [29] studied poly(3-aminobenzoic acid) for H2 production through a H_2_SO_4_ electrolyte with Jph of 0.13 mA cm^−2^. Some studies have been carried out for testing WS2/polythiophene)/Au or Ni/PANI as photocatalytic materials [28,30]. Belabed et al. [31] studied PANI/TiO_2_ for hydrogen generation under artificial light 200 W lamp.

Poly(m-toluidine) (PMT) is a promising polymer with great properties, especially its highly efficient optical absorbance and excellent electrical behavior. Additional advantages are its high environmental compatibility, reproducibility, and stability [24]. There are few studies carried out with this material as a photocatalytic electrode for water splitting.

The previous literature has studied hydrogen generation under water-splitting reactions, but the hydrogen generation rate is still very small. Additionally, the previous studies use a sacrificing agent for the hydrogen generation; sometimes these materials, such as H2SO_4_ or NaOH, with very low or high pH values cause corrosion in the electrodes. Moreover, the previous studies usually use freshwater as a source of hydrogen with the leakage of this water for drinking.

This study prepares PMT, PMT/Au-1 min, and PMT/Au-2 min nanomaterials and characterizes them well using different analytical tools. PMT/Au-2 min is used as an electrode for hydrogen generation from sewage water with high efficiency and low cost. The hydrogen rate is compared with Na_2_S_2_O_3_ electrolytes. The effect of light wavelengths, stability, and temperature are studied. The thermodynamic parameters and hydrogen moles are calculated using different electrolyte sewage water or Na_2_S_2_O_3_.

## 2. Experimental Part

### 2.1. Materials

M-Toluidine (MT) was obtained from VEB Laborchemie Apolda, Germany. Acetone, H_2_SO_4_, Na_2_SO_4_, and Na_2_S_2_O_3_ were purchased from Adwic Company, Cairo, Egypt. ITO glass was obtained from VWR Company, Darmstadt, Germany.

### 2.2. Electropolymerization of MT and Preparation of the Electrode

A potentiodynamic technique in the three-electrode cell was performed in the electropolymerization phase of MT to form PMT, as defined in our previous study [32]. The electropolymerization reaction was carried out on ITO glass (20 Ω) through the anodic polymerization reaction using Potentiostat/Galvanostat Wenking (PGS 95), Hubertusstr, Germany. ITO, PT, and calomel represent the working, counter, and reference electrodes, respectively. During the electropolymerization process, Na_2_SO_4_ and H_2_SO_4_ were used as electrolyte and acid medium, respectively. The electropolymerization reaction was carried out from 0.4 to +1500 mV (in the positive direction) with a scan rate of 40 mVs^−1^. The thickness of the PMT was determined through 10 cyclic voltammetry runs on ITO, then the film was rinsed in water and dried at 60 °C for 12 h.

The sputter coating of the Au was carried out from Au disc (99.99%) using a sputter coating device for 1 and 2 min on the surface of PMT for PMT/Au-1 min and PMT/Au-2 min, respectively.

This electrode was then used for hydrogen generation from sewage water or Na_2_S_2_O_3_ solution, and the results of both electrolytes were compared to each other. The hydrogen generation was carried out from a three-electrode cell, in which the PMT/Au-2 min was the working electrode. At the same time, graphite and calomel were the counter and reference electrodes, respectively.

### 2.3. Characterization Process

The prepared polymer and polymer/Au morphology were characterized using an X-ray diffractometer (XRD) device (X’Pert Pro, Almelo, The Netherlands) Almelo, the Netherlands, worked at 40 mA and 45 kV. FTIR-340 Jasco spectrophotometer, Easton, USA, was used for FTIR analyses. The 1H-NMR measurement (the Varian EM 360 L NMR, Oberkochen, Germany) confirmed the functional groups in the polymer materials. Scanning electron microscopy was used to conduct morphological studies (ZEISS SUPRA Gemini Column, Oberkochen, Germany). Optical analyses of the prepared films were determined using the Shimadzu UV/Vis spectrophotometer (M160 PC, Waltham, MA, USA).

## 3. Results and Discussion

### 3.1. Electropolymerization of M-Toluidine

The electropolymerization process was carried out through the optimization of the conditions of the preparation, represented in the current J_P_ value. This Jp value represents the rate of the reaction [33].

**Figure 1:**
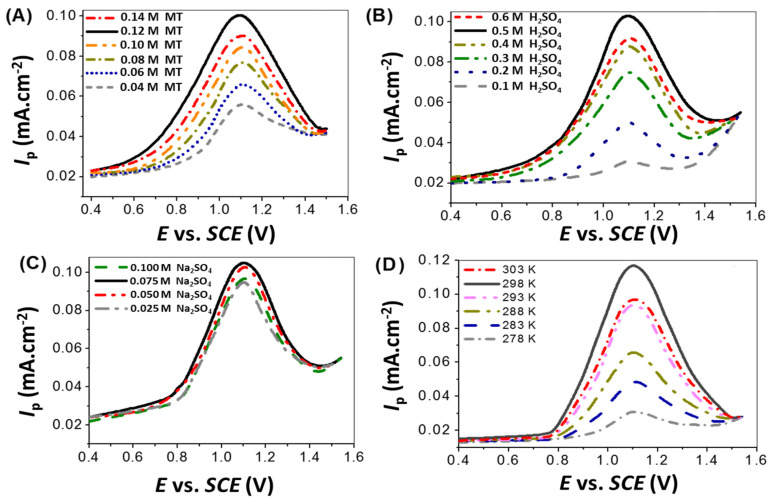
The effect of the concentrations of (**A**) m-toluidine (0.04 to 0.14 M), (**B**) H_2_SO_4_ (0.1 to 0.6 M), (**C**) Na_2_SO_4_ (0.025 to 0.1 M), and (**D**) temperature (278 to 303 K) on the elctropolymerization of m-toluidine.

The effect of the m-toluidine monomer 0.04 and 0.14 M study is shown in Figure 1A at 40 mVs^−1^ and 303 K. The optimum concentration was 0.12 M, with an optimum J_p_ value. After the increase in the concentration to over 0.12 M, more collisions with the monomer take place, which cause the deactivation of the electrode surface [34]; this decreases the J_p_ value to 0.14 M.

In the same manner, the effect of H_2_SO_4_ from 0.1 to 0.6 M is shown in Figure 1B; the optimum concentration of H_2_SO_4_ is 0.5 M. Additionally, the effect of Na_2_SO_4_ from 0.025 to 0.1 Mis shown in Figure 1C; the optimum concentration is 0.075 M. The effect of temperature from 278 to 303 K is shown in Figure 1D; the optimum temperature is 298 K. These conditions confirm that the optimum conditions for the preparation of PMT are 0.12 M m-toluidine, 0.5 M H_2_SO_4_, and 0.075 M Na_2_SO_4_ at 298 K.

### 3.2. PMT and PMT/Au Analyses

**Figure 2:**
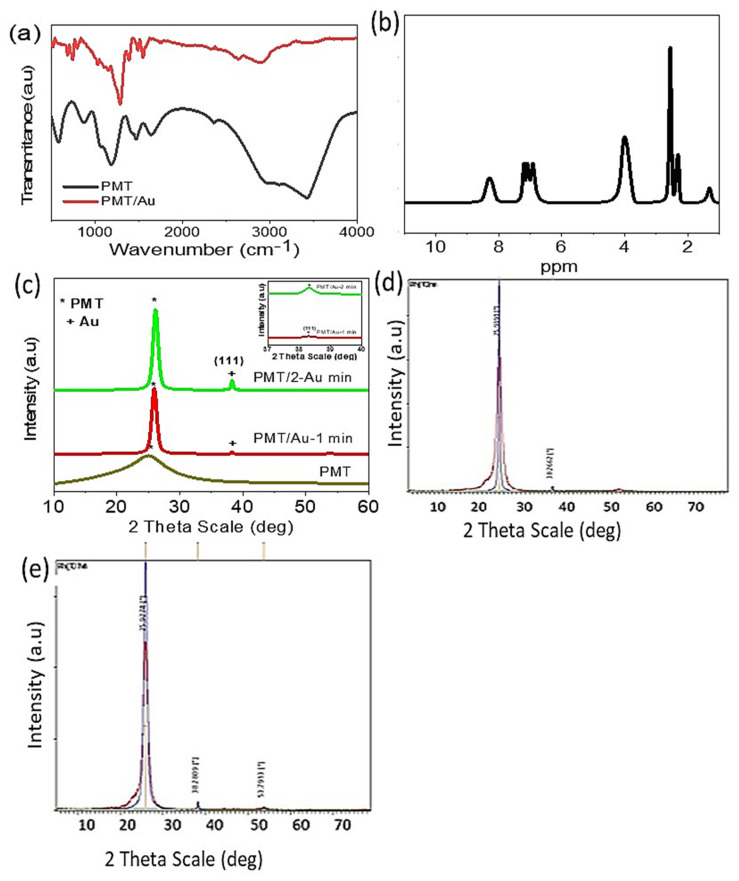
(**a**) FT-IR and (**b**) ^1^HNMR of PMT. (**c**) XRD of PMT, PMT/Au-1 min and PMT/Au-2 min. (**d**,**e**) Standard stick pattern for ITO/PMT/Au-1 min and ITO/PMT/Au-2 min, respectively.

The chemical construction of the PMT nanomaterials is shown in Figure 2a, and the data are summarized in Table 1. From the Figure and the Table, it can be observed that the PMT function groups appeared well, in which the C–H, N–H, and C–N function groups appeared at 3106, 3429, and 1339 cm^−1^, respectively. C=C, benzenoid and quinoid appear at 1465, 1407, and 1631 cm^−1^, respectively. The vibration of C–H in/out of the plan is localized at 1051 and 595 cm^−1^, respectively. After the PANI/Au composite formation, there are shifts in most of the PANI peaks (Figure 2a, red curve), which is related to the interaction between the functional groups and the Au nanoparticles.

For more confirmation of the chemical structure of the PMT, an 1HNMR analysis was carried out, as shown in Figure 2b. The protons related to the methyl and N–H group are located at signals *δ* = 1.23 and 4.07 ppm, respectively. The benzene ring has signals from *δ* = 6.93 to *δ* = 7.27 ppm [35]. The summarized data are shown in Table 2.

To further confirm the chemical structure of PMT, PMT/Au-1 min, and PMT/Au-2 min, XRD analyses were carried out and the results are shown in Figure 2c. The PMT shows a broad peak at 2 *θ* = 25.55, indicating the semi-crystalline nature of the polymer [40,41]. After the Au coating, this peak is more enhanced due to the composite formation; sharp peaks appear in this region with high intensity, which increases from Au coating 1 to 2 min. Moreover, there is an additional peak that appears at 2 *θ* = 38.3° at growth direction (111); this peak increases in intensity for 1 and 2 min coating.

This confirms the growth of the composite with high crystallinity after the Au coating process [41]; this crystalline nature confirms the availability of the composite for water-splitting reaction with high efficiency [42].

Scherrer’s formula [41] (D = 0.9 *λ*/W cos*θ*) was used to calculate the crystal size. This formula depends on the angle (*θ*) in radian, wavelength (*λ*), and the full width half maximum (W). From this formula, we calculated that the crystalline size of PMT is ~15 nm, and this crystalline size increases to 24 and 35 nm, after Au coating for 1 and 2 min, respectively. Additionally, the standard stick patterns for ITO/PMT/Au-1 min and ITO/PMT/Au-2 min are shown in Figure 2d,e, respectively.

**Figure 3:**
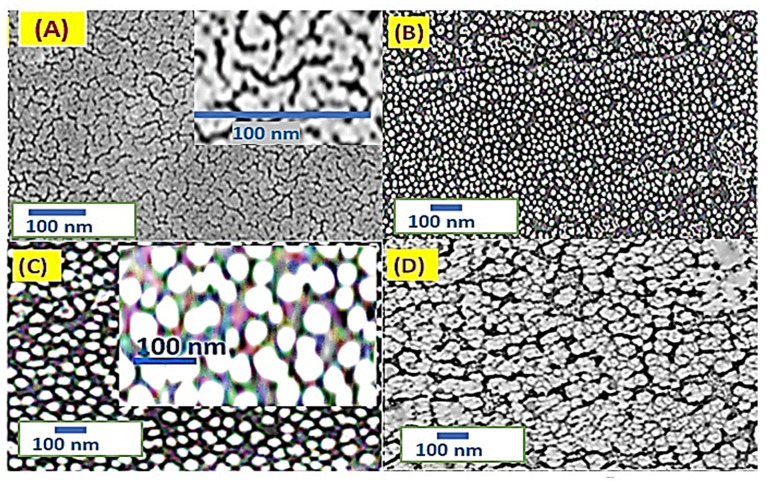
SEM images of PMT for Au coating with times of (**A**) 0, (**B**) 1, (**C**) 2, and (**D**) 3 min.

The morphologies of the prepared PMT, PMT/Au-1 min, and PMT/Au-2 min are shown in Figure 3A–C, respectively. The PMT has a nanopore surface with a smooth lamellar behavior. The diameter of the particles is about 20–30 nm. After Au nanoparticle coating for 1 and 2 min (sputter coating), the porous nature of the surface increased, which is caused by the increase in the active sites of the PMT/Au composite. The average particles sizes of Au nanoparticles for 1 and 2 min are ~15 and 38 nm, respectively. Increasing the Au coating to 3 min (Figure 3D) makes the surface completely blocked with the Au metals. So, these properties prevent the role of the PMT for additional photocatalytic applications.

The PMT/Au-2 min has the optimum features for additional photocatalytic applications from these surface morphology properties.

**Figure 4:**
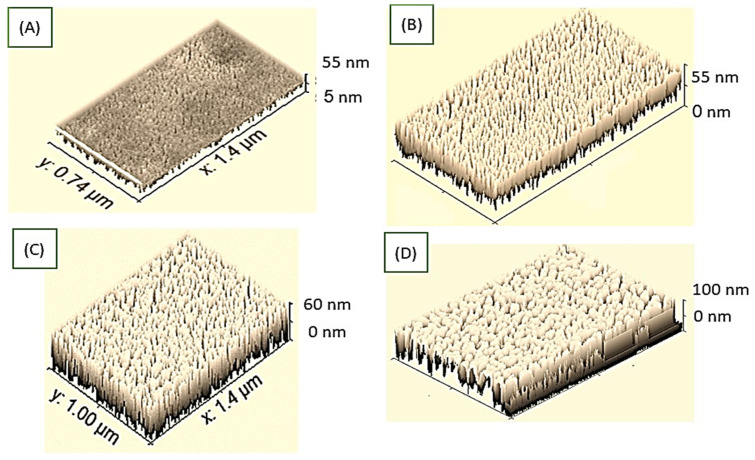
Roughness and cross-section using Image J program for PMT after Au coating for the times of (**A**) 0, (**B**) 1, (**C**) 2, and (**D**) 3 min.

These morphological properties are confirmed using the ImageJ program modeling as shown in Figure 4A–C, for PMT, PMT/Au-1 min, and PMT/Au-2 min, respectively. This program can calculate the cross-section and show the morphology well. From these figures, it can be observed that the PMT morphology is porous with uniform nature; this porosity and roughness increase after the Au coating process for 1 and 2 min. Moreover, the cross-section of the film increases with a Au coating from 1 to 2 min.

By increasing the Au coating to 3 min, the Au nanoparticles completely block the PMT surface. This modeling study confirms the SEM image’s behavior.

**Figure 5:**
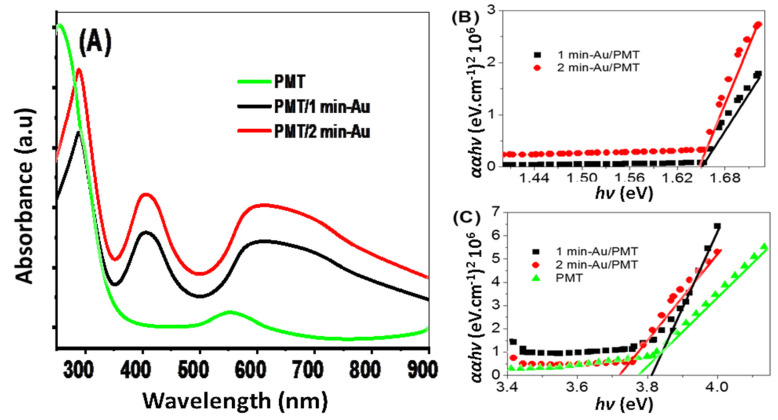
(**A**) The optical absorbance and (**B**,**C**) bandgap values for the PMT, PMT/Au-1 min, and PMT/Au-2 min nanomaterials.

The optical properties of the PMT, PMT/Au-1 min, and PMT/Au-2 min nanomaterials are shown in Figure 5A, and their bandgaps are shown in Figure 5B. From the absorbance curve, it can be observed that the PMT has two absorbance peaks in the UV and Vis regions at 265 and 550 nm, respectively. These peaks are related to the electrons’ transition from band to band and the polymer conjugations chains [41].

After the Au sputter coating for 1 and 2 min, the optical absorbance increases, and the composite has three absorbance peaks, at 295 nm in the UV region and 430 and 605 nm in the Vis region. The peak at 605 nm is related to the plasmonic resonance of Au nanomaterials. After the formation of these three peaks, the composite has peaks that are over most of the optical regions in the UV, Vis, and near IR regions; these peaks have redshifts in wavelengths in comparison with the PMT absorbance.

The Au nanoparticles have a significant role in enhancing the optical properties of the composite. The main property of the Au NPs derives from dimensional confinement (sizes smaller than the wavelength of light), which leads to the alteration of their optical response following the appearance of a phenomenon called “surface plasmon resonance” (SPR), which fundamentally arises from the collective and coherent oscillation of the free conduction electrons in a continuous band structure, due to the resonant excitation caused by the incident photons or electromagnetic radiation [43,44]. This LPSR causes the free electrons of Au to oscillate coherently, creating a strong electric field on the Au nanoparticles. This field is transferred to the neighbor PMT semiconductor polymer for creasing an electron–hole pair [45]. From this process, the electrons are collected on the surface and ready for an additional photocatalytic reaction and to create J_ph_ values. So, the PMT/Au-2 min nanocomposite is qualified to generate photoelectrochemical H2 gas under the water-splitting reaction.

The bandgaps (*E_g_*) for the PMT, PMT/Au-1 min, and PMT/Au-2 min were calculated from Tauc’s equations (Equations (1) and (2)) [46]. These equations are based on the absorbance (*A*), film thickness (*d*), absorbance coefficient (α), light frequency (υ), Boltzmann (*B*), and Planck (*h*) constants [47].

(1) $${\left(\alpha \;h\upsilon \right)}^{2}=B\;\left(h\upsilon -E_{g}\;\right)$$

(2) $$\alpha =\left(\frac{2303}{d}\right)A$$

From Tauc’s equation and Figure 5B,C, it can be observed that the bandgap of ITO/PMT is 3.74 eV for the wavelength 329.12 nm; this value agrees well with the previous literature [48]. After the Au sputter coating and the formation of the PMT/Au-1 min and PMT/Au-2 min, these two composites have bandgaps for the absorbance peaks in the near IR region at 1.64 and 1.63 eV, respectively (Figure 5B). Moreover, another bandgap is also related to the UV region peak at 3.8 and 3.70 eV (Figure 5C) for the two composites, respectively.

These absorbance and bandgap values for the PMT/Au-2 min confirm that this composite has the optimum optical properties compared to PMT or PMT/Au-1 min.

So, the photoelectrode based on this composite is qualified for photocatalytic applications and H_2_ generation through the water-splitting reaction.

### 3.3. Electrochemical Hydrogen Generation

The electrochemical H_2_ generation reaction was carried out through the three-electrode cell, in which the prepared electrode (ITO/PMT, ITO/PMT/Au-1 min, or ITO/PMT/Au-2 min) acts as the working electrode, while graphite and calomel are the counter and reference electrodes, respectively. The measurements were carried out from sewage water to electrolyte without using any additional electrolyte solution; then, these measurements were compared with Na_2_S_2_O_3_ as a reference electrolyte. The measurements were carried out at room temperature (25 °C) under a 400 W artificial light, metal-halide lamp.

**Figure 6:**
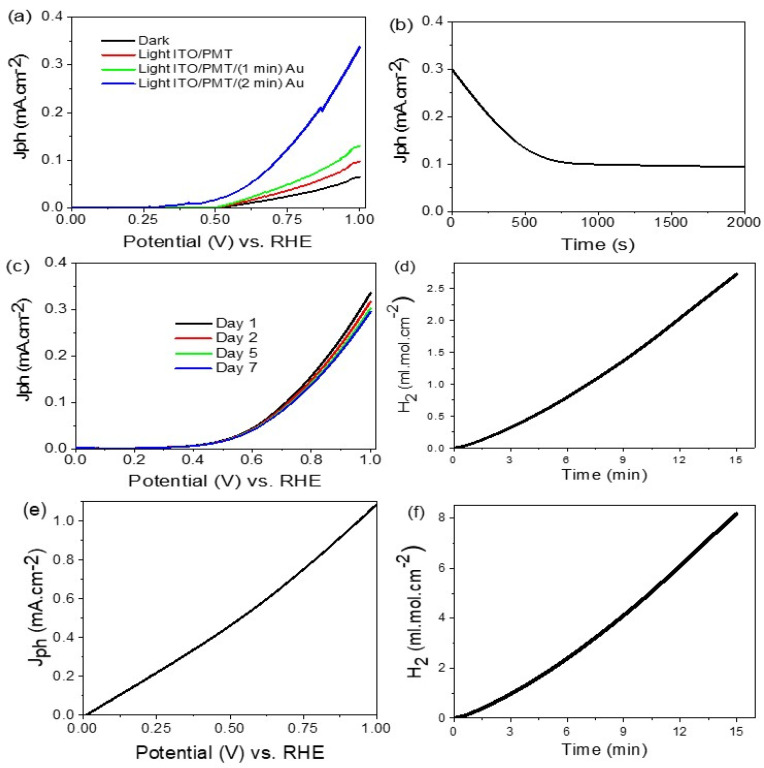
(**a**) Photocurrent voltage curves in dark (black) and under illumination for the ITO/PMT(red), ITO/PMT/Au-1 min (green), and ITO/PMT/Au-2 min (blue) electrodes; (**b**) current–time curve for the ITO/PMT/Au-2 min electrode at 0.75 V; (**c**) current–voltage characteristics for ITO/PMT/Au-2 min electrode under 7 days of illumination with a 400 W metal-halide lamp at 293 K; and (**d**) the evaluated H_2_ moles at room temperature for ITO/PMT/Au-2 min using the Na_2_S_2_O_3_ electrolyte. Sewage water (**e**) current–voltage characteristics for PMT/Au-2 min and (**f**) lifetime.

As shown in Figure 6a, the measurements were carried out in the dark and under light using the Na_2_S_2_O_3_ electrolyte. The J_ph_ values are 0.0.098, 0.12, and 0.33 mA cm^−2^ at 1 V for electrodes ITO/PMT, ITO/PMT/Au-1 min and ITO/PMT/Au-2 min, respectively. This confirms the effect of Au nanoparticles on increasing the J_ph_ values. The Au plasmonic nanoparticles cause an increase in the electrode surface area; this process causes the light capture and generates more electron–hole pairs on the surface of PMT, generating high J_ph_ values for H_2_ generation under the water-splitting reaction.

The small dark current density (J_o_) for the ITO/PMT/Au-2 min electrode is related to the flow of electrons through the PMT and Au junction.

From this comparison using Na_2_S_2_O_3_ as an electrolyte, ITO/PMT/Au-2 min electrode has the optimum behavior for the water splitting and H_2_ generation process with the lowest photogeneration voltage (0.56 V).

The stability of the ITO/PMT/Au-2 min electrode was determined, at an applied bias voltage of 0.75 V, through the relation between the time and the produced J_ph_ values, as shown in Figure 6b. The produced J_ph_ values decrease smoothly from 0.3 to 0.1 mA cm^−2^ on the first time and then become constant until reaching 2000 s. The decrease in the first period is related to the limited corrosion process in the electrode surface under the presence of the electrolyte [49]. The presence of Au nanoparticles protects the PMT layer and decreases the corrosion behavior in this electrode. The stability and reproducibility of the electrode were measured for 7 days through the I–V relation, as shown in Figure 6c. The figure confirms the high stability of the electrode with time in relation to J_ph_, which decreases only from 0.32 to 0.275 mA cm^−2^ from the first to the seventh day, respectively.

The number of hydrogen mole was determined for the electrode, ITO/PMT/Au-2 min, by using the Na_2_S_2_O_3_ electrolyte, as shown in Figure 6d. The hydrogen moles were calculated using Faraday’s law (Equation (3)). This equation depends on the J_ph_ and time (dt) and the Faraday constant (F; 9.65 × 10^4^ C mol^−1^). The produced H_2_ moles are 8.4 mmol/cm^−2^ h; this hydrogen gas evolves as bubbles from the electrolyte, in which the Au nanoparticles enhance the number of moles evolved.

(3) $$H_{2}(\mathrm{moles})=\int_{0}^{t}\frac{J_{\mathrm{ph}}\mathrm{dt}}{F}\cdot 1/2$$

On the other hand, using the sewage water as an electrolyte without using any additional electrolyte is very promising for the H_2_ generation reaction. The chemical construction of the sewage water is shown in Table 3. The J_ph_ value is enhanced after using the sewage water by the electrode PMT/Au-2 min, as shown in Figure 6e. The J_ph_ value reaches 1.09 mA cm^−2^, which is an enhanced value compared with the previous standard electrolyte Na_2_S_2_O_3_ that had a J_ph_ value of 0.33 mA cm^−2^ (Figure 6a). Moreover, the number of the produced H_2_ moles increased highly, as the produced H_2_ moles reaches 33.1 mmol h^−1^ cm^−2^, as shown in Figure 6f.

**Figure 7:**
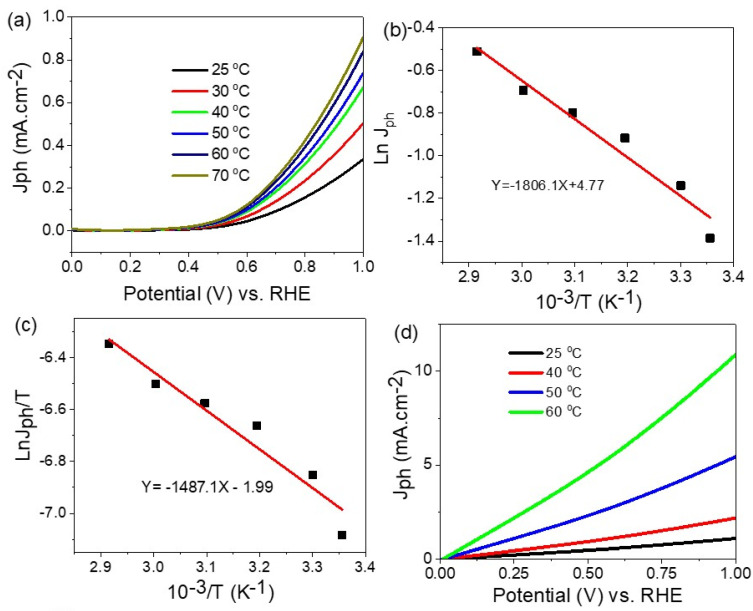
(**a**) The effect of the temperature on the produced J_ph_ value, (**b**) the Arrhenius relation, and (**c**) the Eyring relation for the PMT/Au-2 min electrode using the Na_2_S_2_O_3_ electrolyte. (**d**) The effect of the temperature on the produced J_ph_ value for PMT/Au-2 min electrode using sewage water as the electrolyte.

The effect of temperature of 25–70 °C on the electrode ITO/PMT/Au-2 min for the water-splitting reaction and H2 generation using the Na_2_S_2_O_3_ electrolyte is shown in Figure 7a. The increasing in temperature from 25 to 70 °C causes the J_ph_ values to increase from 0.32 to 0.88 mA/cm^2^, respectively, at 0.9 V. This is related to the role of the temperature in the increase in ion mobility and then the H_2_ generation rate [50].

The activation energy (*E_a_*) can be calculated from the Arrhenius and Eyring equations, Equations (4) and (5), respectively [46,51]. These equations depend on the following factors: the Arrhenius constant (*A*), the universal gas constant (*R*), the temperature (*T*), the rate constant (*k*), the Boltzmann constant (*B*), and the Planck constant (*h*). From the Arrhenius equation and Figure 7b, the activation energy (*E_a_*) is 31.49 KJ mol^−1^.

(4) $$k=Ae^{-E_{a}/RT}$$

The t ΔH* and ΔS* values are calculated from the Eyring equation and Figure 7c, in which the values are 114.49 Jmol^−1^ and 160.46 JK^−1^ mol^−1^, respectively:

(5) $$k=T\cdot \frac{kB}{h}\cdot \;e^{\Delta S/R}\cdot e^{-\Delta H/RT}$$

On the other hand, after using the sewage water as an electrolyte, there is more enhancement in the produced J_ph_ values in comparison with the standard Na_2_S_2_O_3_. The increase in temperature from 25 to 60 °C causes the J_ph_ values ro increase from 1.09 to 11.2 mA cm^−2^, respectively. This confirms the superiority of sewage water as an electrolyte for the water-splitting and H_2_ generation reaction.

**Figure 8:**
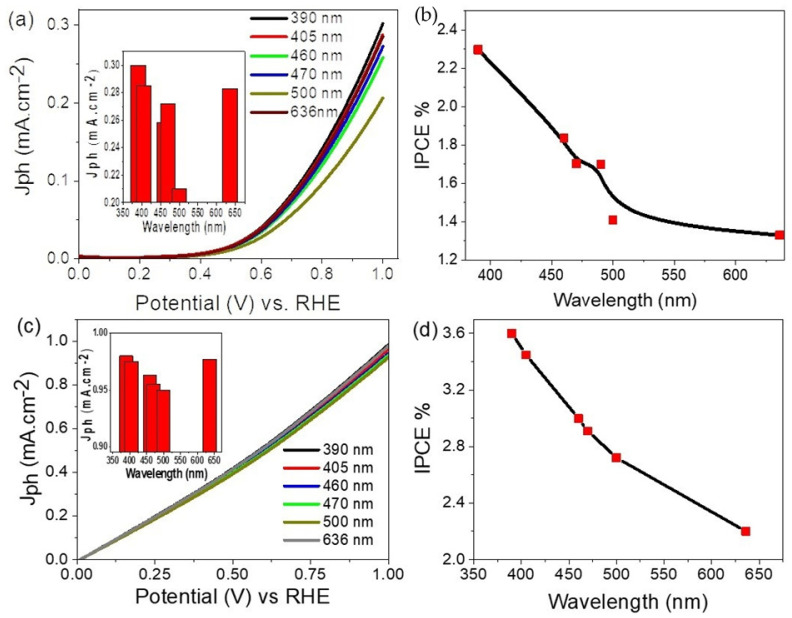
(**a**) The response of the PMT/Au-2 min electrode under different monochromatic light from 390 to 636 nm using electrolytes (**a**) Na_2_S_2_O_3_ and (**c**) sewage water. The *IPCE* through using (**b**) Na_2_S_2_O_3_ and (**d**) sewage water electrolyte.

The effect of monochromatic light wavelengths between 390 and 636 nm on H_2_ generation using the ITO/PMT/Au-2 min electrode is shown in Figure 8a. From this figure, the produced J_ph_ values decrease from 0.30 to 0.20 mA cm^−2^, increasing the wavelengths from 390 to 500 nm, respectively. Then, the J_ph_ increases again until it reaches 0.28 mA cm^−2^ at 636 nm. The wavelength 500 nm has the minimum J_ph_ value; this behavior is matched well with the optical absorbance spectrum in Figure 5A. Moreover, the good values of J_ph_ in the broad wavelength region confirm the solar absorption for the ITO/PMT/Au-2 min and H_2_ generation in these light regions. Moreover, the Au nanoparticles play a good role in enhancing light absorption, and they overlap in the response of Au and PMT nanomaterials to produce J_ph_ values at lower potential.

Figure 8b represents the photon-to-current conversion efficiency (*IPCE*) for the electrode ITO/PMT/Au-2 min under the light for the water-splitting reaction and H_2_ generation using the Na_2_S_2_O_3_ electrolyte. This *IPCE* can be calculated using Equation (6) [52]. This equation depends on the J_ph_, wavelength (λ) and light intensity (*ρ*). The *IPCE* value is 2.3% at 390 nm. These values are compared with the previous literature as shown in Table 4.


(6) $$IPCE\;\left(\%\right)=1240\cdot \;\frac{J_{\mathrm{ph}}}{\lambda \;\cdot \;\rho }\cdot \;100$$

Using sewage water as an electrolyte for H_2_ generation using the electrode ITO/PMT/Au-2 min, there are more enhancements in the produced J_ph_ values as shown in Figure 8c. The variation of the produced J_ph_ values with the monochromatic light has the same behavior by using Na_2_S_2_O_3_ as an electrolyte. The J_ph_ values decrease from 0.98 to 0.91 mA cm^−2^, increasing the wavelength from 390 to 500 nm. Then, the J_ph_ value increases to 0.97 mA cm^−2^ at 636 nm. The *IPCE* for the H_2_ generation is 3.6%. These values are greater than the previous values by using the Na_2_S_2_O_3_ electrolyte.

### 3.4. Mechanism

The prepared electrode PMT/Au-2 min mechanism for H2 generation from Na_2_S_2_O_3_ or sewage-water electrolyte was carried out using two steps; interfacial charge transfers take place due to the electron–hole transfer. In addition to the localized surface plasmonic resonance (LSPR), this process causes the oscillation of the electron on the surface of the semiconductor material. These two phenomena can appear well through the optical analyses (Figure 5A,B) and the electrochemical curve and H2 moles produced (Figure 6a–f). Under the light incident, there is an electron transfer from the LUMO to HUMO for the PMT [60]; then, these electrons oscillate on the surface of the PMT and transfer the energy from the Au to PMT. The wide range of absorbance related to the PMT/Au composite increases the electron generation on the surface that is finally collected on the PMT surface under the LSPR process [61]. More electrons are generated with this high electromagnetic coupling between Au and PMT. Then, more H2 generation reactions occur [62]. The electron transfer depends on the work function of Au and the electron affinity of PMT.

**Figure 9:**
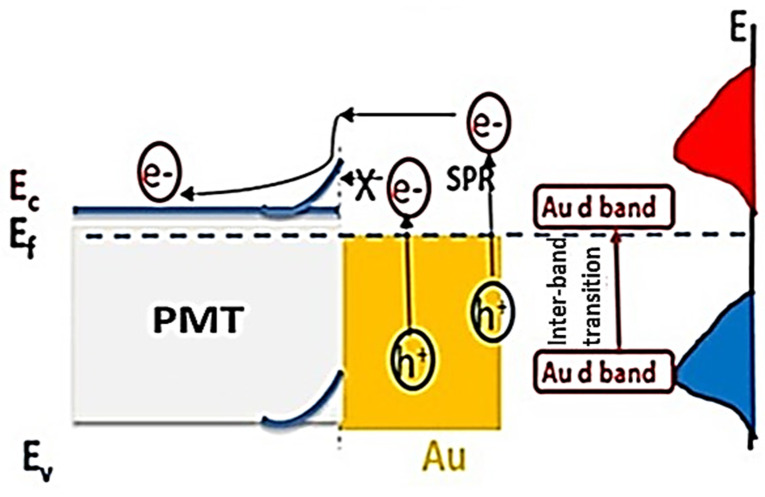
The mechanism of water splitting reaction using the PMT/Au-2 min electrode.

Moreover, the presence of the Schottky barrier limited the electron transfer under the presence of the internal electric field. This barrier push transfers the photoexcited electrons. At the same time, it prevents electron–hole pair recombination. This process causes an enhancement in the produced J_ph_ and then water splitting for the H_2_ generation reaction [63]. The hot electrons pass to the PMT, while the cold electrons still suffer the internal barrier, as shown in Figure 9. Finally, these hot electrons are collected on the PMT surface for water splitting and H_2_ generation reaction.

## 4. Conclusions

PMT preparation was carried out by the electropolymerization of m-toluidine on the ITO using the anodic polymerization method. Then, Au under different sputter coating times (0, 1, 2, and 3 min) was deposited on the PMT, in which the ITO/PMT, ITO/PMT/Au-1 min and ITO/PMT/Au-2 min electrodes were prepared. The full characteristic analyses were carried out to confirm the chemical structure and morphology of the prepared nanomaterials. From the different analyses, the electrode ITO/PMT/Au-2 min has the optimum optical properties to be applied as an electrode for water splitting and H_2_ generation. The thermodynamic parameters were calculated for the H2 production from water using Na_2_S_2_O_3_ as an electrolyte, in which *E_a_*, ΔS*, ΔH* values were 31.49 KJ mol^−1^, 160.46 JK^−1^ mol^−1^, and 114.49 J mol^−1^, respectively. The electrode has high stability and reproducibility for H_2_ generation reaction, in which the J_ph_ was decreased from 0.32 to 0.27 mA/cm^2^ during the 7 days. The H_2_ generation under sewage water splitting was carried out using the PMT/Au-2 min with high *IPCE* 3.6% at 390 nm, and the produced H_2_ moles evolved were 33.1 mmol h^−1^ cm^−2^. These results were compared with the previous data of the Na_2_S_2_O_3_ electrolyte with maximum *IPCE* 2.3% and H_2_ moles of 8.4 mmol/cm^2^.h.

We will continue this research by working on the synthesis of an electrochemical cell for sewage water splitting directly. Sewage water can be used as electrolyte inside the cell directly for H_2_ gas production that is used as fuel inside homes, factories, and companies. This cell will be promising for renewable energy production, especially in remote regions, such as deserts and spacecraft.

## Figures and Tables

**Table 1:** The FT-IR analysis of the PMT nanomaterial.

Band Position (cm^−1^)	Assignment
3429	N–H group [35,36,37]
3106	Aromatic C–H group
2950	Methyl group stretching [32]
2357	Adsorbed H_2_O or CO_2_ from the atmosphere [38]
1465 and 1407	C=C benzenoid [32]
1631	C=C quinoid
1339	C–N vibrations
1051	C–H in-plane
595	C–H out of plane
879	Aromatic rings para-disubstituted

**Table 2:** The summary of the HNMR analysis.

Chemical Shift (ppm)	Assignment and Structure
1.23	The proton of the methyl group [39]
2.56	DMSO proton (solvent) [35]
2.3	Adsorbed H_2_O
4.07	Singlet signal for NH proton [35]
6.93 to 7.27	The protons of the benzene ring [39]

**Table 3:** The chemical construction of sewage water.

Material or Element	Concentration (mg/L)
Phenols	0.015
F^−^	1.0
Al^3+^	3.0
NH_3_	5.0
Hg^2+^	0.005
Pb^2+^	0.5
Cd3^+^	0.05
As^3+^	0.05
Cr^3+^	1.0
Cu^2+^	1.5
Ni^3+^	0.1
Fe^3+^	1.5
Mn^2+^	1.0
Zn^2+^	5.0
Ag^+^	0.1
Ba^3+^	2.0
Co^2+^	2.0
Other cations	0.1
Pesticides	0.2
CN^−1^	0.1
Industrial washing	0.5
Coli groups	4000/100 cm^3^

**Table 4:** *IPCE* and J_ph_ values of the present work compared with the previous literature.

Electrode Materials	Electrolyte	*IPCE*% (390 nm)	J_ph_ (mA cm^−2^)
BiFeO_3_ [53]	Na_2_SO_4_	--	0.16
Au/Pb(Zr,Ti)O_3_ [54]	Na_2_SO_4_	--	0.06
CdSe/TiO_2_ nanotube electrode [55]	NaOH	0.45	0.13
Fe_2_O_3_/sodium dodecyl sulfonate electrodes [56]	NaOH	2	0.05
SnO_2_/TiO_2_ [57]	Na_2_S_2_O_3_	--	0.4
ZnO/Ag [58]	Na_2_SO_4_	0.5	1
PrFeO_3_ [59]	Na_2_SO_4_	1.2	0.13
ITO/PMT/2 min-Au (present work)	Na_2_S_2_O_3_	2.3	0.3
ITO/PMT/2 min-Au (present work)	Sewage water	3.6	0.98

## Data Availability

The data that support the findings of this study are available from the corresponding author upon reasonable request.
